# Abstracts of Papers Read at the Meeting of the State Medical Association at Dallas, May 8-10, 1917

**Published:** 1917-06

**Authors:** C. R. Hannah

**Affiliations:** Dallas, Texas


### Abstracts of Papers Read at the Meeting of the State Medical Association at Dallas, May 8-10, 1917.

### VOMITING OF PREGNANCY—CAUSE AND TREATMENT.

BY DR. C. R. HANNAH, DALLAS, TEXAS.

Vomiting of pregnancy is always the result of gestation. It only occurs in the pregnant woman.

Two symptoms of pregnancy, cessation of menstruation and morning sickness, are well advertised and universally known to all doctors and to most all child-bearing women.

Very frequently the patient makes her diagnosis of pregnancy by these two symptoms. It is well for the physician to give more than a passing notice to these symptoms, for from the history of the menstruation he may be able to find that nausea is often present, if not always, at each period. This knowledge may be very essential when treating vomiting of pregnancy. Then, too, morning sickness, while occurring in more than half of all pregnancies from the sense of nausea to that of vomiting, may continue into the very worst form and yet be called "morning sickness."

A complete clinical history of every case of pregnancy should be had to assist in diagnosing the form of vomiting of pregnancy. This is very essential. To neglect this important feature may often mislead one and render the treatment absolutely useless.

The cause of vomiting of pregnancy is probably yet unknown, and, until it is, the treatment must and will be more or less symptomatic.

Williams, probably, gives the most logical and practical analysis of this subject. To me, it is the best. He states that totally different pathological conditions may be produced by identical clinical symptoms, and that to differentiate and properly classify the form of toxemia of pregnancy must be qualified to isolate the specific cause or recognize the pathological lesions present.

Vomiting, other than "morning sickness," may occur any time during the day or night and, too, it may continue over a long period. This alone should differentiate vomiting of pregnancy from "morning sickness." Happily, however, this form of vomiting is most frequently relieved by improvement in the patient's hygiene, small doses of laxatives and rest in bed.

In Williams' Monograph of 1906, also in this text-book, he subdivides this subject into three divisions, namely, reflex, neurotic and toxemic. The reflex variety is the result of some abnormality, usually of the reproductive organs, yet, it is true, a pathological lesion in any organ may be the root of all the evil. This variety alone should teach us the value of a thorough clinical history, combined with a physical examination.

The neurotic type may manifest itself in conjunction with the reflex, the neurosis being the result of a hidden pathological lesion, such as ulcer of stomach, chronic appendicitis, heart lesion or any disturbance that influences and disorganizes the nervous system. Here, again, a complete clinical history proves invaluable, and by it unhygienic and unnatural influences will be found and an attempt to improve them can be immediately instituted.

The toxic variety is evidently the direct result of a metabolic disturbance. It is this type that usually proves fatal. In the first two named types, little if any pathological changes are found, while in the toxic, necrosis of the central portion of the lobule of the liver occurs and the periphery remains intact. The pathology is very similar to that of acute yellow atrophy, yet the clinical symptoms differ.

We should bear in mind that pernicious vomiting of pregnancy or toxic vomiting differ from pre-eclampic toxemia or eclampsia. It is true they may have some clinical symptoms in common, yet the pathological lesions differ in that the liver necrosis of the central portion of the lobules occurs in toxic vomiting, while in eclampsia the lesion is one of thrombosis and begins in the periportal spaces.

The renal change is one of distinction and usually limited to a necrotic condition of the epithelium of the convoluted tubules, which frequently occludes their lumen. These lesions are doubtless due to the toxic processes, since the burden of relief is borne by these organs, striving to neutralize the poisons.

A condition of acidosis should never be overlooked or forgotten in any of the types, and more especially is this true when toxins are thought to be the cause. Starvation and an underlying pathological lesion uncovered are contributory causes of this condition. To say the least, we must be able to recognize and cope with this complication.

*Treatment.*—In the reflex type, correct any malposition of the uterus, remove tumors, or a troublesome appendix, and in brief treat and restore to normal function any offending organ.

In the neurotic type, ascertain home surroundings, improve her hygiene, and apply and put into practice rigid suggestive therapeutics. The success of this treatment depends upon the co-operation of the family and the personality and firmness of the doctor. Dilatation and application to the cervix is nothing more than mental suggestion, and is good psychology. Elimination by the bowels and free use of water per anum are excellent.

In the toxic variety, much has been tried and yet we still are groping our way in the dark. Drugs with which we are all familiar "have been weighed in the balance but found wanting." Adrenalin in some cases proves valuable. Hirst gives a series of cases in which he used corpus luteum. From these cases he claims improvement and believes it should be given a trial. Personally, I might have had success in one of the three cases in which I used it, yet at times I doubt it. DeLee does not place much faith in it.

For the past few years I have used this treatment:

1. Insist upon the patient taking her breakfast in bed and remaining there for one or two hours.

2. Eat whatever she wants at any time during the day and even immediately after vomiting.

3. Make a thorough vaginal examination, and, if normal, let her alone.

4. Give large doses of sodium bicarbonate (drachm one to eight ounces of water) six times per day.

Dr. J. M. Newman, of New Orleans, advises me "that the most important of all the treatments combined is the use of horse serum." Proceed as follows: "Inject in the lumbar region subcutaneously (just like using any other serum) 1 c.c. of the normal horse serum and wait three or four hours for any anaphylactic reaction, should same appear within this time—do not proceed further; if, however, no reaction takes place—then inject the remaining 9 c.c.

"In desperate cases I give the 5 per cent glucose solution intravenously (one quart) and allow the serum to run in at the same time.

"The patient usually shows marked improvement after twenty-four hours, and the progress of convalescence goes rapidly. If, however, the improvement is only slight, I would advise repeating the serum in three or four days again, provided, of course, everything else is normal."

